# Supplementary material for: Sclerostin as Regulatory Molecule in Vascular Media Calcification and the Bone–Vascular Axis
Source: Toxins (Basel). 2019 Jul 21;11(7):428. doi: 10.3390/toxins11070428 (PMC6669501; doi:10.3390/toxins11070428)
Supplement: Supplementary file 1 [file toxins-11-00428-s001.pdf]

Supplementary Materials: Sclerostin as Regulatory Molecule in Vascular Media Calcification and the Bone–Vascular Axis

Annelies De Maré, Stuart Maudsley, Abdelkrim Azmi, Jhana O. Hendrickx, Britt Opdebeeck, Ellen Neven, Patrick C D'Haese and Anja Verhulst

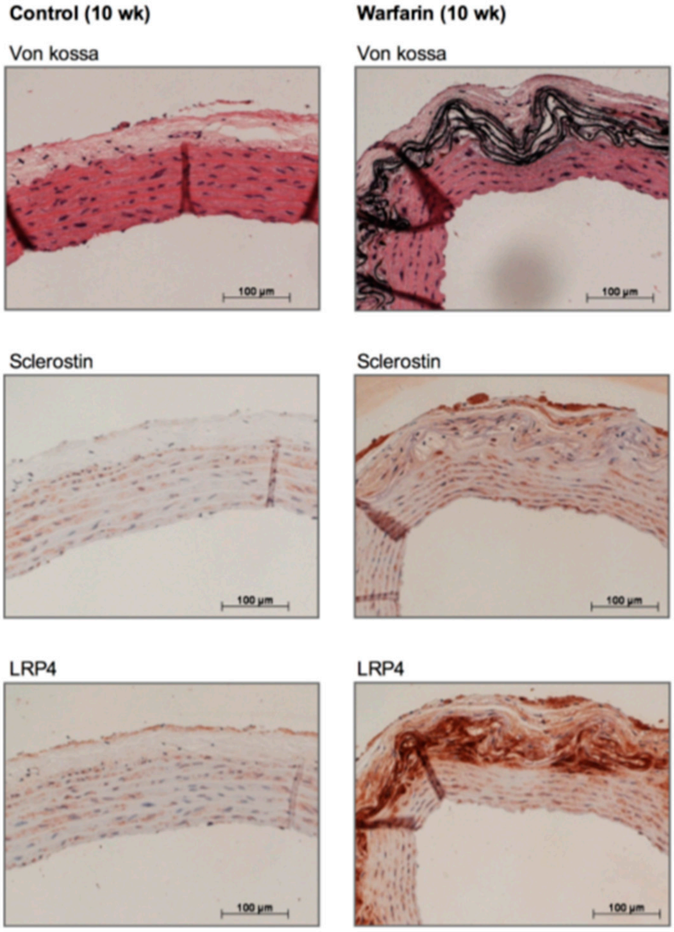

Figure S1: Consecutive tissue sections, stained with von Kossa and immunostained for sclerostin and LRP4. Tissue sections of a control rat (left) and a 10-week warfarin-treated rat (right).
